# Wernicke Encephalopathy Due to Hyperemesis Gravidarum in Pregnancy: A Case Report

**DOI:** 10.7759/cureus.2991

**Published:** 2018-07-17

**Authors:** Vikash Talib, Shazia Sultana, Ahmed Hamad, Uzair Yaqoob

**Affiliations:** 1 Accident & Emergency, Jinnah Sindh Medical University (SMC), Karachi, PAK; 2 Obstetrics and Gynecology, Ziauddin University, Karachi, PAK; 3 Medical Student, Ziauddin University, Karachi, PAK; 4 Surgery, Jinnah Postgraduate Medical Centre, Karachi, PAK

**Keywords:** hyperemesis gra, wernicke's encephalopathy

## Abstract

Wernicke’s encephalopathy (WE) is a life-threatening acute or sub-acute neurological emergency characterized by ataxia, confusion, nystagmus, and ophthalmoparesis caused by thiamine deficiency. It was first described in 1881 by Carl Wernicke with alcohol being the most common cause. We present a rare case of a 35-year-old pregnant female who presented to our emergency department with a history of vomiting and loose motions for two weeks. She later developed fever, confusion, slurred speech and blurring of vision. Magnetic resonance imaging (MRI) of the brain revealed typical lesions of WE. She was immediately treated with thiamine and her symptoms improved in a few days.

## Introduction

Wernicke’s encephalopathy (WE) is a neurological emergency characterized by ataxia, confusion, nystagmus, and ophthalmoparesis caused by thiamine deficiency. It was first described in 1881 by Carl Wernicke [1]. Alcoholism is the most common cause but it can also result from an unbalanced diet, vomiting, anorexia nervosa, untreated inflammatory bowel disease, and bariatric surgery [2]. The relation between WE and hyperemesis gravidarum was described in 1939 by Sheehan [3]. Here, we describe the case of a 35-year-old pregnant female with WE due to hyperemesis gravidarum and discuss its possible cause, clinical presentation, diagnostic imaging, and treatment.

## Case presentation

A 35-year-old pregnant patient at 16 weeks presented to our emergency department with symptoms of vomiting, loose motions, confusion, fever, slurred speech, and blurring of vision for four days. The patient was treated with intravenous fluids for hyperemesis gravidarum in another hospital without much improvement during the previous 14 days. On examination, the patient was awake, restless, confused, responding verbally, following one step commands on repeated verbal stimuli, neck soft, restricted extraocular movement, incomplete ophthalmoparesis with bilateral lateral and medial gaze paresis and ocular bobbing in upward vertical gaze. Both plantars were down-going and the patient was moving all four limbs simultaneously. The tone was normal in all four limbs. Power was 4/5 in both upper limbs and 0/5 in lower. The muscle stretch reflexes were 4/5 in all four limbs.

**Figure 1:**
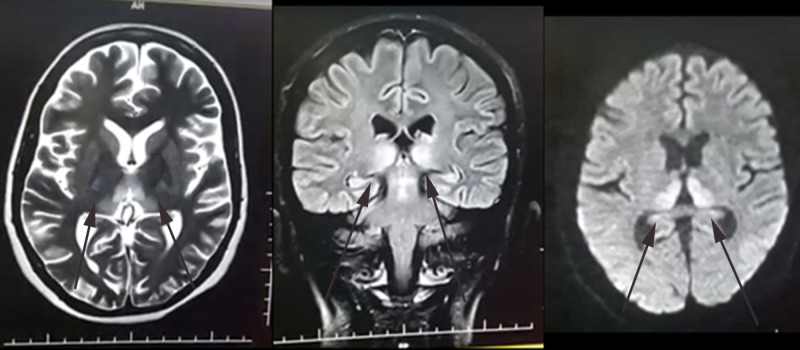
Magnetic resonance imaging (MRI) of the brain showing hyper-intense and diffusion restricted areas in the peri-tectal region and bilateral medial thalami

Laboratory investigations showed hemoglobin of 13.2, total leucocytes count of 11.2 with 40% lymphocytes. Urine detailed report showed a leucocyte count of 10 and was nitrite positive. Other tests including platelet count, urea, and creatinine, electrolytes all came out normal. Ultrasound was ordered which showed single alive intrauterine gestation of 16 weeks. Magnetic resonance imaging (MRI) showed hyper-intense and diffusion restricted areas in the peri-tectal region and bilateral medical thalami (symmetrical) suggestive of WE as shown in Figure 1.

The patient received thiamine 100 mg two times daily for three days then 100 mg once daily till complete resolution of symptoms; the patient also received other vitamin B supplements during hospitalization. The patient’s condition improved dramatically over the next few days and she was discharged on parenteral nutrition.

## Discussion

The body stores about 25 mg to 30 mg of water-soluble vitamin thiamine (B1) which is good for about 18 days. The daily requirement is 0.4 mg/1000 kcal per day which increases in pregnancy to 1.5 mg/day [4]. Deficiency of thiamine pyrophosphate, being an important co-factor for enzymes in the pentose phosphate pathway, affects multiple tissues particularly those with high thiamine turnover which includes neural parenchyma resulting in cell necrosis or apoptosis [5].

WE in pregnancy generally occurs in women who are malnourished, increased demands of pregnancy. Hyperemesis further depletes thiamine stores [6]. Our patient was also malnourished and appeared underweight. However, WE is uncommon in pregnancy but hyperemesis gravidarum is pretty common. WE generally occurs during 14-18 weeks gestation after two to three weeks of vomiting [5] which was the case with our patient who presented at 16 weeks gestation with history of vomiting for two weeks.

The diagnosis of WE is primarily clinical. In his operational criterion for the identification of WE, Caine et al. proposed that WE is diagnosed if any two of the following four signs exist: ophthalmoplegia, ataxia, altered mental status, and malnourishment [7]. Nystagmus is the most common ocular sign and confusion is the most common presenting symptom [3] which was also evident in our patient. The diagnosis is confirmed by an MRI brain which shows symmetric high T1, T2, and T2 flair signal intensities in the mammillary body, medial thalamus, periventricular, and periaqueductal regions. The sensitivity and specificity of brain the MRI is 53% and 93% for the diagnosis of WE respectively [8]. Our patient's MRI showed T2 hyper intense and diffusion restricted areas in the peritectal region and bilateral medial thalami which was symmetrical confirming the diagnosis of WE.

Polyneuropathy can also be present in patients with WE which can include sensory, motor, and autonomic dysfunction. It can also include paralysis and anesthesia. Axonal degeneration and demyelination are the two most important mechanisms responsible for polyneuropathy due to decreased adenosine triphosphate (ATP) supply [9]. Our patient presented with decreased power 0/5 in both lower limbs which reflects that polyneuropathy is more severe in distal limbs.

The gold standard treatment is to replace thiamine which results in the resolution of symptoms in a few hours to a few days depending on the severity of the disease. If untreated, it can progress to Wernicke-Korsakoffs (KS) syndrome resulting in more chronic symptoms such as anterograde amnesia which takes more time to resolve after treatment [10]. Therefore, diagnosis and treating as fast as possible will result in less severe disease.

## Conclusions

WE is an uncommon and life-threatening neurological disease complicating pregnancy in the setting of hyperemesis. MRI is the investigation of choice. Timely and serious management of vomiting in pregnancy and replacing thiamine stores help treat acute neurological symptoms and can prevent both maternal and fetal morbidity.
